# “You don’t expect a miracle to happen soon”: a qualitative study of psychosocial support needs of caregivers of children with disability in Eastern Uganda

**DOI:** 10.1186/s12887-026-06906-3

**Published:** 2026-04-23

**Authors:** Airi Imamura, Ronald Tenywa, Kathryn Jones, Peter Waiswa, Gertrude Namazzi, Atsumi Hirose

**Affiliations:** 1 School of Public Health, Faculty of Medicine, Imperial College London, London, UK; 2 School of Medicine, University College Dublin, Dublin, Republic of Ireland; 3 School of Social Sciences, Makerere University, Kampala, Republic of Uganda; 4 School of Public Health, Makerere University, Kampala, Republic of Uganda

**Keywords:** Neurodevelopmental conditions, Coping mechanisms, Psychosocial support, Support needs, Post-infancy children, Primary caregivers, Eastern Uganda, Intervention

## Abstract

**Background:**

Globally, an estimated 53 million children under 5 years live with disabilities. The majority live in low- and middle-income countries. The government of Uganda legislated the Uganda National Social Protection Policy in 2015 and the Persons with Disabilities Act in 2020 to foster an inclusive and non-discriminatory environment. However, challenges and implementation gaps persist. Previous studies highlighted the caring burden on caregivers of infants with disabilities. We explored the lived experiences of parents of children with developmental disabilities in Uganda, focusing on the effects of children’s disabilities on daily life, the strategies they use to cope with these challenges and their unmet psychosocial support needs. By capturing these caregiver perspectives, the study seeks to inform inclusive programme designs and policies, particularly in low-resource settings.

**Methods:**

This qualitative study was conducted at an outpatient paediatric neurology clinic annexed to a regional referral hospital in Eastern Uganda in March 2024. In-depth interviews were held, using an interview guide, with nine primary caregivers of children between 1 and 6 years old with developmental disabilities to capture insights into their experiences and support needs and two healthcare professionals to obtain their perspectives. Data were transcribed, coded, and thematically analysed using a framework approach.

**Results:**

Five themes were identified: ‘struggles’, ‘information’, ‘faith’, ‘accepting the situation’ and ‘looking for a solution’. Mothers faced emotional and financial ‘struggles’ caring for children with developmental disabilities and prejudice extending from spiritual beliefs. They found comfort in ‘faith’ and were determined to seek ‘information’ and a cure for the conditions; however, a large knowledge gap existed due to the lack of accurate and trustworthy information, which opened further because of their isolation from the community. Many learned to cope through ‘accepting the situation’, but continued ‘looking for a solution’ to children’s conditions. Healthcare providers also called for health system changes to provide further support.

**Conclusions:**

Better access to information, education, social care, and counselling services and cross-sector collaboration are needed to remove the stigma and enhance caregivers’ quality of life. The development of holistic cross-sector support interventions are required.

**Supplementary Information:**

The online version contains supplementary material available at 10.1186/s12887-026-06906-3.

## Background

Globally, 317 million children and adolescents live with developmental disability [1]. Developmental disabilities include a range of underlying health conditions that affect the developing nervous system during the prenatal period, infancy or childhood, and cause impairments in motor, cognitive, language, behaviour and / or sensory functioning. The health conditions include those listed in the International Classification of Diseases 11th Revision (ICD-11) under neurodevelopmental disorders (such as autism spectrum disorder, developmental learning disorder, developmental motor coordination disorder and attention deficit hyperactivity disorder) [2], as well as a group of congenital conditions such as Down syndrome or conditions acquired at or after birth, such as cerebral palsy and epilepsy [1].

As the previous Millennium Development Goal (MDG) 4, launched in 2000, prioritised child survival over optimal growth [3], the global burden of developmental disabilities remained unchanged since 1990, with approximately 53 million under-5 children living with developmental disabilities in 2016, and the majority living in low- and middle-income countries [4]. The Sustainable Development Goals, which succeeded the MDGs in 2015, sought to address these gaps by including disability-specific targets and adopting a more holistic approach to child development. However, people with disability are disadvantaged across many spheres of their lives. To keep the promise of ‘leaving no one behind’, attention must now be paid to supporting 250 million children in low- and middle-income countries (LMICs) at risk of not reaching their developmental potential [5] including those with developmental disabilities.

The government of Uganda ratified the UN Convention on the Rights of Persons with Disabilities (UNCRPD) in 2008 [6] and legislated the Uganda National Social Protection Policy in 2015 and the Persons with Disabilities Act in 2020 [7]. The laws ensure the rights of individuals with disabilities and focus on accessibility, participation, capacity building, awareness raising, prevention and management of disabilities, care and support, socio-economic security, research, communication and budgeting [8] to foster an inclusive and non-discriminatory environment. These policies are intended to endorse the implementation of the social model of disability [9] – developing interventions that target disabling environments, social barriers and cultural attitudes in addition to medical and therapeutic interventions. However, significant challenges and implementation gaps persist: the social model may not be fully grounded in the lived experiences of disabled people and often overlooks the interaction of individual impairments, socioeconomic circumstances and cultural values [10], thereby underrepresenting many disabled populations in need.

To overcome this implementation gaps, parents, supported by a wider community, who play the central role in providing effective nurturing care to children with developmental disabilities [11, 12] could advocate for the children in removing the social barriers addressed by the social model of disability [10]. However, they often face significant emotional, physical and financial challenges that are exacerbated by the lack of psychosocial support [13]. Few studies have been conducted to explore the lived experience of caregivers when raising a child with a developmental disability in LMICs [14–22]. For example, caregivers of children with disabilities in Kenya described high emotional distress, need for information, social isolation and discrimination as well as lack of access to healthcare and educational services [15, 20], while research from South Africa has highlighted stigma and limited family-centred support systems [19, 21]. Despite these insights, there is still limited evidence in relation to how the caregivers’ psychosocial support needs change over time. In Eastern Uganda, prior studies have explored the experiences of caregivers of infants with neurodevelopmental disabilities [18, 22] and highlighted the benefits of focusing on education and services in optimising socio-neurodevelopment. However, the support needs of parents with older children with developmental disabilities, who may face additional unique challenges arising as the children grow and have different caring needs, remain unexplored. Therefore, this study aims to explore the impact of disabilities of children between 1 and 6 years old, in the post-infancy and early childhood, on the daily lives of primary caregivers, focusing on their coping mechanisms and psychosocial support needs, to inform future policies in Uganda and similar settings.

## Methods

This study was conducted at an outpatient paediatric neurology clinic (‘the clinic’) in Eastern Uganda, annexed to a regional referral hospital serving a population of 4.5 million people.

On weekly clinic open dates in March 2024, during morning clinic registration, the data collection team (RH, GN and AI) approached and invited primary caregivers meeting the eligibility criteria (Table 1) to individual interviews.

**Table 1:** Eligibility criteria of primary caregivers

Inclusion criteria	Exclusion criteria
They are primary caregivers of children aged between 1 and 6 years old	Their children are less than 1 year old or more than 6 years old
They are primary caregivers of children with clinical diagnoses of neurodevelopmental conditions associated with developmental disabilities [1] verified by the registry of the study clinic	Their children do not have clinical or working diagnoses of neurodevelopmental conditions associated with developmental disabilities [1]
They are able to give consent	They are not able to give consent
They are over the age of 18 years old	They are less than 18 years old
Their children are not in emergency or inpatient care	Their children need emergency or inpatient care

We initially employed convenience sampling, approaching caregivers in the waiting area. However, on the second day, we transitioned to purposive sampling to ensure demographic balance and to recruit children of both genders with various developmental disabilities as more participants expressed interest. Upon obtaining consent from caregivers, children’s clinical records were reviewed to confirm eligibility. In addition, a purposive sample of two healthcare providers (HCPs) meeting the criteria (Table 2) was recruited from different healthcare professions.

**Table 2:** Eligibility criteria of healthcare providers

Inclusion criteria	Exclusion criteria
They regularly provide care to children with neurodevelopmental conditions associated with disabilities [1]	They do not regularly provide care to children with neurodevelopmental conditions associated with disabilities [1]
They work as healthcare professionals at the study clinic	They do not work as healthcare professionals at the study clinic

Separate interview guides for primary caregivers and HCPs containing open-ended questions both explored challenges in caregivers’ lives across multiple domains, including well-being and quality of life (QoL), social care, financial burden, psychological distress, family beliefs and community participation and their expectations and perceptions towards the support they receive (Additional files 1 and 2).

The interview guides were initially developed in English, informed by relevant literature [13–15, 18, 23, 24] and prior qualitative research experience of members of the research team in similar settings. The draft English versions were reviewed collaboratively by the research team and pre-tested by AI with a small number of peers in the UK to assess clarity, flow and relevance of questions. Minor revisions to wording and sequencing were made following this initial pre-testing.

The caregiver interview guide was subsequently translated into Lusoga by RT. The translated guide was then pre-tested by AI and RT with a small number of caregivers attending the clinic who were not included in the final analysis. This stage of pre-testing focused on cultural appropriateness, sensitivity of language nuances and relevance and depth of questions in the local context. Further minor refinements were made to the phrasing and sequencing of questions to improve comprehension and facilitate richer responses. Overall, the development and refinement of the interview guide followed an iterative process to ensure suitability for the study population and context.

Using the finalized caregiver interview guide, RT conducted individual face-to-face in-depth interviews with caregivers in Lusoga within hospital premises, typically lasting 30–45 min. AI conducted interviews with HCPs in English outside of their working hours on clinic premises to obtain their perspectives on the caregivers’ psychosocial support needs. In-depth interviews were chosen as the primary method of data collection due to their suitability for exploring sensitive and complex topics. This approach allows participants to express their lived experiences in their own words, leading to collection of rich data with deep insight into their struggles, coping mechanisms and interactions with healthcare and social systems. It is particularly appropriate given that the aim of this study was to understand caregivers’ psychosocial support needs within their specific sociocultural contexts [25]. Although a summary was not provided at the end of each interview, we created a poster several months after data collection to present the findings to the clinic and staff members as a form of informal community validation, ensuring the key messages resonated with their experiences before publication. All interviews were audio-recorded.

As we approached the final interviews, we observed that thematic saturation had been reached, with no new themes emerging. Previous research suggests that code saturation in qualitative studies can be achieved with small sample sizes, typically ranging between 9 and 17 interviews, particularly when the study has a narrow focus and involves a relatively homogenous participant groups [26], which applies to our study.

Interviews with primary caregivers were translated into English and transcribed by RT. Interviews with HCPs were transcribed by AI. All transcripts were read multiple times for familiarization, then open-coded manually line by line and cross-checked to ensure consistency. Initial codes were developed inductively and grouped into broader categories based on recurring patterns by AI and KJ. These categories were indexed and charted into a matrix based on key areas explored in the interview guides. Thematic analysis using a framework approach [24, 25, 27] was employed, and the findings were interpreted to derive themes using current literature and refined through team discussions. Throughout the coding and analysis process, AI received regular guidance from KJ, an experienced qualitative researcher, ensuring alignment with the research objectives and supporting methodological rigour.

### Statement of reflexivity 

This study was conducted as part of AI’s dissertation for the intercalated BSc in Global Health, undertaken during her medical degree, and supervised by AH. AI developed a specific interest in developmental disabilities and support systems in early childhood, particularly in low-resource settings. Motivated by this interest and by prior work led by GN, AI and AH specifically sought a collaboration with the Ugandan researchers (PW, GN and RT) and received guidance from them in designing and conducting the study. AI then conducted fieldwork in Uganda in close collaboration with RT and under the supervision of GN. 

The research team acknowledges that our positionalities – across age, gender, ethno-racial and educational backgrounds – shape our perspectives and interactions throughout the research process. In embracing self-reflexivity, AI recognises her perspective as a young student researcher affiliated with academic institutions in high-income countries. We are aware that despite our best intentions, the privilege conferred by our educational backgrounds and affiliations with Western institutions could inadvertently perpetuate perceptions of patronisation, insults and imperialism associated with colonial legacies and existing power imbalances. In trying to critically interrogate in this research, we were committed to prioritise direct observations and approach participants with humility to minimise preconceived notions.

However, we acknowledge that none of the authors are caregivers of children with developmental disabilities in Uganda. Our positionalities may limit the understanding of their circumstances and carry the risk of imposing data interpretations shaped by privilege. Moreover, with all the authors being multilingual and/or having international research backgrounds, we are well cognisant of the potential misunderstanding and loss of meaning in translation, thereby affecting data validity. To address these complexities, we have adopted a reflexive and collaborative approach throughout the research process. AI, AH and KJ remained in ongoing dialogue with GN and RT to ensure cultural and contextual sensitivity in both data collection and analysis. Coding and interpretation were informed by this partnership with relevant local and international literature. 

Finally, we remain mindful of the broader impact of our research on the communities we study, striving to uphold ethical principles and mitigate the infliction of adverse effects. Our aspiration is to conduct research that is not only academically rigorous but also equitable and inclusive. 

## Results

A total of 11 interviews, including nine primary caregivers and two HCPs, were analysed. All the primary caregivers who participated in the study were female (Table 3), henceforth referred to as ‘mothers’. We used pseudonyms when referring to their quotes. Similarly, their children with developmental disabilities are hereafter referred to as ‘children’. The two HCPs were part of a multi-disciplinary team running the outpatient neurology clinic.

**Table 3:** Participant demographics

	Children (n = 9)	Primary caregivers (n = 9)
Age	Median 2 (1-6)	Median 35 (21-41)
Gender	Male: 6	Male: 0
	Female: 3	Female: 9
Age at diagnosis	Median 1 (0-3)	
Type of underlying health conditions	Epilepsy: 6	
	Cerebral palsy (CP): 4	
	Autism spectrum disorder (ASD): 2	
	Down syndrome: 1	
Marital status		Married: 7
		Separated: 1
		Widowed: 1
		Divorced: 0
Education level		No education: 1
		Primary education (Primary 5-7): 3
		Secondary education (Senior 1-4): 5

A table showing the demographic details of primary caregivers and their children participating in the study. The median and the range are noted for age, and the number of participants reporting each gender, type of neurodevelopmental condition, marital status and educational level are reported

**Fig. 1:**
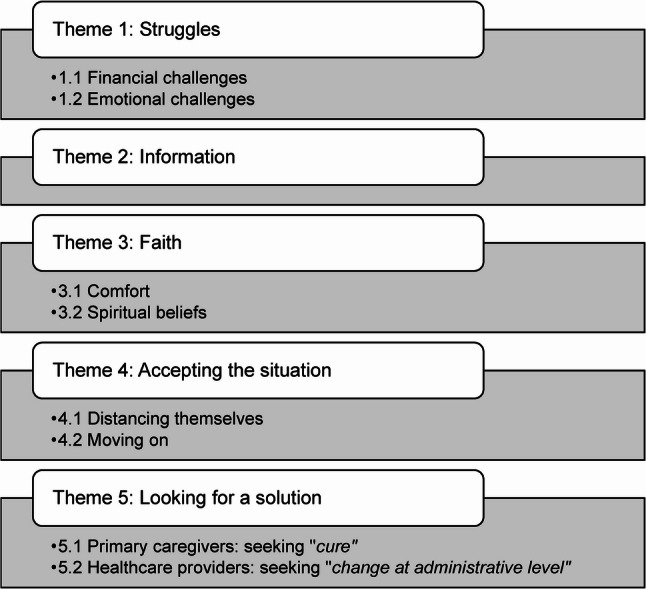
Summary of themes and subthemes. A diagram listing five themes and one or two relating subthemes occurring in each theme explored in the study. The subthemes written in bold are the subthemes selected to be explained in this study

Five main themes were identified: (1) struggles, (2) information, (3) faith, (4) accepting the situation and (5) looking for a solution. Themes and sub-themes can be found in Fig. 1.

### Struggles

Rural, mostly poor mothers financially struggling to provide their child’s care and older siblings’ educational needs faced distress and emotional struggles in their daily lives, aligning with the HCPs’ observations.

#### Financial challenges: *“We have spent all the money on this child”* (Alice, Mother of a 3-year-old)

Mothers described themselves as ‘not doing so well’ financially and considered themselves ‘poor’. HCPs also highlighted financial challenges as the foremost concern; they described most mothers who came from rural areas as ‘fac[ing] poverty’ and ‘struggl[ing] with getting transport’ to the hospital.

Some mothers said: ‘Most times, we fail to get what to feed the children’. This occurs despite the mothers wishing to provide their children with sufficient food.

*‘These children want to feed well*,* so*,* if your income is not stable*,* you cannot sustain such a child’.* (Emily, Mother of a 1-year-old)

Mothers reported spending most of their money on coming to the hospital, making it difficult to meet basic needs in some cases and causing more struggles when they also needed to buy medicine. Although the hospital offers medication during visits as a ‘public’ healthcare service institution, the HCPs referred to limited resources and often ‘run[ning] out of stock’ of medication.

*‘Sometimes they just prescribe medicine*,* and we have to go and buy it outside the hospital.’* (Alice, Mother of a 2-year-old)

Although the mothers understand the hospital’s struggles, they find it ‘painful’ when they ‘lack money to go and buy it’ as their children then cannot take medication to improve their conditions.

Moreover, mothers reported that the financial burden extended to the siblings of the children as many caregivers ‘fail to pay school fees for other children’, leading to them having to forgo education or switch schools. Many are already selling their properties and incurring loans, but these measures are insufficient to meet their needs. Hence, financial support for basic needs and access to healthcare and medication tops the list of mothers’ most desired and required support.

#### Emotional challenges: *“Life becomes very tough”* (Charlotte, Mother of a 1-year-old)

Many mothers expressed overwhelming distress and exhaustion exacerbated by the relentless demands of caring for their children without respite. During difficult times, they reported grappling with negative thoughts.

*“I find myself quarrelling alone*,* speaking to myself*,* I sometimes regret why God gave me this child. At times I ask myself what wrong I committed for God to give me such a punishment.”* (Fiona, Mother of a 1-year-old)

During consultations and interviews, mothers frequently displayed raw emotional reactions when discussing their daily struggles.

Recalling initial experiences post-diagnosis, they recounted feeling ‘scared’ and ‘overwhelmed’ by uncertainty, reporting they ‘lost senses’ out of fear. What saddens them and makes them want to ‘give up’ is seeing other children born at the same time ‘talking and walking normally’.

HCPs emphasised the necessity of ‘regular counselling’ to ‘involve them, talk to them, take them through what the child is going through and try to make them understand the needs of the child’. Charlotte (Mother of a 1-year-old), feeling overwhelmed by the demands of everyday life and her child’s special needs, recounted receiving counselling from an organisation affiliated with a local university, which significantly alleviated her stress. However, the majority of mothers reported relying solely on informal psychological support from family and friends.

#### Information *“I don’t know the condition that is disturbing my child”* (Harper, Mother of a 4-year-old)

Mothers reported challenges in obtaining accurate information and emphasised the critical need for reliable and comprehensive information about their children’s conditions. The hospital visit was often driven by a quest to deepen understanding of their children’s conditions and available care services.

*“I came here at the hospital to seek advice.”* (Grace, Mother of a 3-year-old)

Health education sessions aimed to address informational gaps. However, many mothers reported they lacked a comprehensive understanding of their child’s conditions – what they are, what caused them and how to treat them – and expressed frustration over not receiving a thorough explanation of their children’s diagnosis, hindering their ability to provide adequate support despite a strong desire to ‘learn how to care’.

*“They have never told me the real problem this child has.”* (Charlotte, Mother of a 1-year-old)

Moreover, some recounted experiences where healthcare workers ‘have not even heard about it’, particularly at local healthcare institutions attended before. One HCP mentioned the presence of effort put in by the staff from the clinic visiting communities to educate the local healthcare workers. However, definitive diagnoses often remained elusive until referral or arrival at the clinic.

Outside healthcare settings, some mothers reported learning from radio or television programmes designed to ‘educate people how to care for children with disabilities’. Informal networks formed during waiting times for consultations or through physiotherapy sessions provided opportunities for information exchange.

*“I met many children with the same condition*,* and some are worse.”* (Danielle, Mother of a 1-year-old)

For many, interactions at the hospital and seeing other children with similar challenges served as sources of reassurance and solidarity, noting how encounters ‘strengthened’ their hearts. They were ‘satisfied’ with services provided by the hospital, albeit with a desire for more information about sources of social support, including details on ‘school and education for children with disabilities’.

### Faith

Faith serves as a vital coping mechanism providing comfort during difficult times, yet spiritual beliefs served as a trigger of discrimination, leading to psychological distress and social isolation.

#### Comfort: *“It was God who gave me this child”* (Brooke, Mother of a 2-year-old)

Mothers, identifying as Christians or Muslims, stated they ‘pray to God’ for protection and healing. Some expressed a wish for ‘a person that God allows’ or ‘a person whom God puts’ to support them.

*‘I don’t know if God will bring me that person who will help and look after my child.’* (Fiona, Mother of a 1-year-old)

Some mothers credited prayers for contributing significantly to their child’s recovery and ‘thank God’ for improvements in their children’s conditions.

For many, prayers make them feel ‘relieved’ when ‘overwhelmed’ and ‘feel[ing] it is too much’.

*‘In case I feel oppressed by anything*,* I kneel down and pray for God and Allah to help me because he is the one who gave me this child.’* (Brooke, Mother of a 2-year-old)

Some interpreted their children’s conditions as part of ‘God’s plan’ and ‘left everything in his hands’ to aid coping. Attending church or mosque provided an opportunity for solace and community connection.

#### Spiritual beliefs: *“It seems the child was bewitched; you jumped demons when you were pregnant.”* (Fiona, Mother of a 1-year-old)

Mothers reported that cultural beliefs attributed developmental disabilities in children to spiritual causes including clan issues, twin-related matters, God’s blessings, or bewitchment. Such interpretations often led to blame directed at mothers.

*‘Some people were saying we did not perform rituals after producing the twins.’* (Harper, Mother of a 4-year-old)

Both the HCPs and mothers noted prejudice occurred within families and communities extending from spiritual beliefs. Some recounted instances of family and friends distancing themselves, citing concerns of their children’s conditions being ‘infectious’ diseases. Isabella’s (mother of a 4-year-old) husband decided to ‘run away and abandon’ the entire family, claiming ‘the cause of sickness’ came from her family’s lineage. These responses reflect the social stigma mothers experienced as a result of spiritual attributions. Instances of social exclusion were also reported.

*‘The neighbours don’t want my child to play with their children; they claim my child is going to infect their children with this condition.’* (Grace, Mother of a 3-year-old)

*‘People disrespect me because of this child*,* many people beat him*,* everyone was abusing him.’* (Harper, Mother of a 4-year-old)

Mothers expressed this as ‘painful’, exacerbating their feelings of depression and isolation.

### Accepting the situation

Acceptance played a crucial role in combatting challenges. Self-isolation in response to changed relationships with family, friends and community members helped to move on and focus on caring for the child.

#### Distancing themselves: *“People are not the same”* (Alice, Mother of a 3-year-old)

Many mothers highlighted profound shifts in the attitudes of their family, friends and community members following the birth of children with developmental disabilities. These changes in social attitudes notably eroded a sense of trust among affected mothers, leading to sentiments such as ‘people are no longer trustworthy’. One example involves instances of relatives and friends declining requests to look after the children as they were ‘scared’ or ‘disgusted’ with them. This reluctance is also evident in some fathers.

*“[My husband] used to tell me he is not the father of the child*,* he was quarrelsome.”* (Emily, Mother of a 1-year-old)

While some mothers expressed ‘trust’ in husbands who continued to provide financial, physical, and psychological support, others experienced the husbands complaining, abusing or reminding the mothers about ‘his advice of an abortion’. Many mothers expressed their husbands are ‘tired’ and ‘fed up’ and less involved in seeking care for their children.

Another common experience among mothers was the loss of friendship.

*“People will rumourmonger around about you.”* (Fiona, Mother of a 1-year old)

Many ‘stopped sharing secrets’ beyond a few trusted family members and friends, who can ‘keep confidentiality’ and ‘don’t discriminate’ and primarily relied on their families, particularly their own mothers, for financial, physical, psychological and social support.

Ultimately, the loss of trust and feelings of guilt drive mothers to distance themselves from others as a self-defence mechanism against further emotional harm. Harper (Mother of a 4-year-old) decided to attend a different church as her congregation demanded a level of concentration her child could not sustain. Many mothers described themselves as a source of support, claiming ‘no one’ supports them physically, financially and socially.

#### Moving on: *“You don’t expect a miracle to happen soon but only to live as they are”* (Charlotte, Mother of a 1-year-old)

Mothers recounted learning to confront challenges by striving to ‘stay calm’ and ‘become firm’. When faced with rumours or negative remarks from family or community members, they described they ‘don’t listen’ and instead ‘focus on saving the life of [their] child’.

*‘I had a lot of stress and depression at the beginning then the issue of not working*,* but I have coped with them because I have accepted my child.’* (Brooke, Mother of a 2-year-old)

Many emphasised deciding to ‘accept and care for the child’ – a significant emotion-driven coping mechanism. Some reached this resolution upon hearing words from family members and friends encouraging them to ‘stay strong’ and ‘continue with the care’ of their children. The mothers described these supportive individuals ‘sympathise’ with them, ‘encourage’ them to seek help from healthcare professionals and ‘console’ them that their children will receive the necessary care. Others found acceptance through their sense of maternal responsibility.

*‘I came to accept that this is my child*,* I am the one who produced him*,* I have to live with him the way he is.’* (Isabella, Mother of a 4-year-old)

Many mothers now take pride in dignified care for their children, recognising how some parents in their community ‘feel ashamed of having children with those disabilities’ and hide them. Towards the end of the interviews, some mothers left messages for other struggling mothers, advising them to ‘handle the children with care’, to not ‘mistreat the child’ and to ‘love their children as they are’.

### Looking for a solution

Mothers were desperate for any possible remedy for their children’s developmental disabilities, while the healthcare providers were also ‘looking for a solution’ that exceeded the capabilities of the clinic alone, hoping for systemic changes.

#### Primary caregivers: seeking *“cure”* (Alice, Mother of a 3-year-old)

Interviews with mothers underscored their quest for ‘any support that can help [their] children to improve’. Recalling the period before seeking help from healthcare facilities, some mothers expressed their willingness to pursue any avenue to alleviate their child’s condition.

“*I could not give up on my own child so I went wherever they told me, and I could do whatever they advised me to do.*” (Harper, Mother of a 4-year-old)

HCPs highlighted the desperation of mothers, attributing their willingness to even explore spiritual healings or witchcraft to a significant ‘knowledge gap’ regarding ‘chronic’ developmental disabilities.

*“Explaining to them that this is permanent sometimes is too much.”* (HCP2)

Many mothers described either attempting or being encouraged by family or community members to seek treatment from traditional or spiritual healers. Reflecting on her experience, Harper (Mother of a 4-year-old) reported, ‘There are times I regret going to traditional healers, and I wish I only went to the professional healthcare workers’ as there was ‘no improvement’ despite emotional and financial investments.

Numerous mothers initially sought care from public hospitals within their residential districts. Disappointed by lack of progress they either received referrals or self-referred to the clinic in the hope of receiving better care. If there is still ‘no improvement’, HCPs reported some mothers go ‘missing’ from hospital records.

#### Healthcare providers: seeking *“change at administrative level”* (HCP2)

To improve existing services, HCPs expressed a need for increased medication supplies, a larger staff complement and partners or volunteers who can contribute by establishing facilities and providing equipment. They emphasised the necessity for a holistic approach to providing chronic care for children, which is challenging to manage within the current system already operating at its maximum capacity as a public hospital. They described further enhancements requiring ‘administrative-level’ changes, where systems for hospital service organisation, including supply provision, staff education and guidelines, are reviewed and adjusted.

## Discussion

### Main findings

This study highlights the emotional struggles faced by mothers caring for children with developmental disabilities, who are determined to seek information and a cure. However, the reality is a lack of trustworthy information, and a large knowledge gap, which opens further due to mothers distancing themselves from the community. With a lack of awareness about available support and past mistreatment within families and communities, the current support heavily relies on faith and healthcare available at the neurology clinic and little else.

The WHO Nurturing Care Framework suggests caregivers are most able to provide nurturing care when they are emotionally, financially and socially secure [11]. The pre-school period is a crucial window of opportunity to consolidate the gains from early intervention and sustain or improve developmental trajectories [28]. However, the mothers in this study faced various stressors, including physical, financial, emotional, stigmatic and nursing burdens such as intensive daily care and supervision required by their children [18], which could become a barrier to nurturing care.

Having a child with a severe health condition increases the likelihood of divorce or separation [29]. Paternal abandonment is common among children with developmental disabilities in Uganda [30] which places financial and emotional burdens solely on mothers, making care-seeking and treatment challenging.

Spiritual beliefs and misconceptions regarding the aetiology of developmental disabilities further perpetuate psychological challenges. Many caregivers in LMICs experience stigmatisation and blame assignment for having children with disabilities [31], preventing them from feeling empowered to reach out for support. Stigma is a barrier to healthcare-seeking and a cause of difficulties in relationships and community participation [32].

Many mothers also cling to the hope of a cure without realising the chronic nature of these conditions, a finding similar to an Ethiopian study [14], where caregivers believed their child’s condition was curable. This misunderstanding, compounded by spiritual beliefs, delays appropriate interventions.

### Implications and recommendations

There needs to be a conducive environment with access to information, resources and entitlements through empowered communities, supportive services and enabling policies [11]. We suggest three priorities to create a conducive environment for nurturing care.

1. Potential role of faith-based organisations

Our study highlighted mothers’ acceptance as a coping mechanism and many found solace in faith, viewing their children as gifts from God, similar to other cultures [15]. Religious beliefs have the potential to support caregivers, evidenced by similar findings from studies in Ethiopia [14], the Philippines [16] and Kenya [15]. In Uganda, faith-based organisations have contributed to reducing HIV/AIDS-related stigma and discrimination through prevention, care and advocacy efforts [33] and could play a role in helping to remove stigma of developmental disabilities and improve psychological support for caregivers.

2. Potential role of public healthcare institutions and NGOs

Many mothers initially sought care from local healthcare facilities (one through referral by the village health teams (VHTs)) and non-profit organisations (NGOs), underscoring their pivotal role as initial entry to the healthcare system. Both also offered physiotherapy, reducing the financial burden of transportation costs for already financially constrained families. Further efforts should focus on improving access to rehabilitation services, allowing caregivers to only seek care from neurology clinics for most severe cases. Despite the substantial number of NGOs in the study area, none provide services not already available at public healthcare institutions. There is a need to mobilise resources for advocacy, prevention, routine developmental screening, and treatment of developmental disabilities. Education and training of healthcare professionals to identify and manage these conditions is crucial as well as addressing risk factors, particularly for cerebral palsy, considering its high prevalence in the study area [34].

3. Development of holistic and targeted support interventions

The WHO nurturing care framework is universal and was developed mainly to address the problem of an estimated 250 million children at risk of not realising their developmental potential. However, effective nurturing care of children with disabilities is demanding and complicated because of the stress and the burden borne by the caregivers. Targeted support from across health, education and social protection sectors is needed. In both high-income countries (HICs) [35] and LMICs [23, 36], support and funding for educational provision and access to professional health services are limited. Yet, care coordination interventions enhancing care integration among families and paediatric service providers across health, education and social sectors have been successfully implemented in HICs [37] including England where community paediatricians can refer parents to support for wider determinants of health [38–40]. A similar intervention would be more difficult in LMICs due to fragmented governance and funding sources [41], yet efforts should include developing a system where patients are referred to social and financial services. The VHTs, the first contact point for health-related issues connecting communities and health facilities, could be trained on support provision and availability of referrable services.

### Strengths and limitations

Our multidisciplinary team obtained a detailed description of the experiences of the caregivers of children with disabilities building on previous research [18] to provide new insight.

However, analysing translated transcripts may have failed to capture cultural nuances. In addition, despite purposive sampling, the majority of participants were caregivers of children with epilepsy or cerebral palsy, likely due to its high prevalence in the study area [34], potentially over-representing the experiences of these conditions. The interviewed mothers sought healthcare, thus the caregivers who did not seek healthcare or ones who faced further access barriers are under-represented. The severity of developmental disabilities could not be assessed due to the unavailability of clinical tools, potentially overlooking differences in opinions arising from different severity.

## Conclusions

This study exploring the struggles faced by primary caregivers, their coping mechanisms and their support needs suggests a need to empower them with information, education and social care and counselling services. Collaboration with local leaders to remove stigma-related issues could help create an environment for nurturing care. Collaboration across health, education and social protection sectors are warranted.

## Supplementary Information

Additional file 1.

Additional file 2.

## Data Availability

The datasets generated and/or analysed during the current study are not publicly available due to assuring participant anonymity but excerpts may be available from the corresponding author on reasonable request.

## Abbreviations

HCPs: Healthcare providers
HICs: High-income countries
ICD-11: International Classification of Diseases 11th Revision
LMICs: Low- and middle-income countries
MDGs: Millennium development goals
QoL: Quality of life
