# Supplementary material for: “You don’t expect a miracle to happen soon”: a qualitative study of psychosocial support needs of caregivers of children with disability in Eastern Uganda
Source: BMC Pediatr. 2026 Apr 23;26:533. doi: 10.1186/s12887-026-06906-3 (PMC13245006; doi:10.1186/s12887-026-06906-3)
Supplement: Supplementary file 1 — Additional file 1. [file 12887_2026_6906_MOESM1_ESM.pdf]

Interview guide for caregivers of children with disability in semi-urban eastern Uganda

Respondents

Primary caregivers of children with neurodevelopmental disorders (NDDs) who attend the outpatient paediatric neurology clinic of Jinja Regional Referral Hospital in Jinja City, eastern Uganda every Wednesday during the 2-week interview period in February and March 2024

Objective

To explore in-depth whether and how carer's daily lives are affected by pre-school children's disabilities, their coping mechanisms and to understand their psychosocial support needs

Introduction

We wish to explore support needs among primary caregivers. Through these interviews, we would like to identify challenges in carers' lives across multiple domains (e.g. family structure and resources, family problems, social support, child problems, acute stress, sibling problems, family well-being, illness behaviour and family quality of life) and to establish their support needs.

Questions

Section 1: Family structure and demographic information

1A. Are you a primary caregiver? – Yes. / No.

1B. Please tell us who lives in your child's home and what is their relationship to him/her.

Relationship to patient	Age	Sex
1. Patient (your child visiting the clinic today)		
2. Person being interviewed (you)		
3.		
4.		
5.		
6.		

1C. What is your relationship / marital status? (Please check one.)

- ○ Single
- ○ Married / partnered
- ○ Separated / divorced
- ○ Widowed
- ○ Other (describe)

Section 2. Well-being and quality of life

2A. Please tell me about the reasons why you have been referred to this clinic.

- I understand that your child is referred to this clinic because he/she has a certain disability. Do you mind telling us what type? What help are you hoping to get from the clinic visit today?

2B. How satisfied / happy are you with your quality of life nowadays (in terms of happiness, relationships with family members and friends and well-being)?

Prompts:

- Do you feel that you have good relationships with your friends and family members?
- Do you have anyone whom you can trust and confide in?
- Do you often feel overwhelmed by the demands of everyday life? How well do you manage to cope with them?
- Do you feel that you have been able to do what you would like to do in your life?

Section 3. Social care

Who can you count on to provide the support and help?

Prompts:

- Who could help you if you need help to look after your child(ren)?
- Who could help you if you need advice about your child's special needs related to their disability?
- Who could help you if you need financial support?
- Who could help you if you need help with everyday tasks or chores?
- Have you been able to find information on social support for families who have children with disability?
- What support and help have you received from in and outside of your family?
- You may not have asked for support, but has someone offered to provide you support?

Section 4. Financial burden

How is your family doing financially?

Prompts: Do you have some financial problems? Do you find it difficult to meet your basic needs such as buying food or paying rent or mortgage?

Section 5. Psychological distress

Now I would like to ask you how you are doing emotionally/psychologically? How have your daily lives been affected since you found out that your child may have a developmental delay or disability?

Prompts: How has your sleep been affected? Have you been able to do normal day-to-day tasks as before? Do you feel sad or depressed? Have you experienced any physical change such as tiredness, breathing difficulties or stomach pains?

Section 6. Family beliefs

6A. Has your family life been affected by your child's disability? If so, how?

6B. Has your child's disability affected your relationship with your friends, relatives and neighbours? If so, how?

6C. Has your child's disability / developmental delay affected your other children? If so, how?

6D. In your experience or community, are there specific beliefs or explanations regarding the causes of disability / developmental delay? If yes, please tell me about them.

Prompts: Have these beliefs changed the perception of disability / developmental delay within your family or your community?

Section 7. About taking part in your community …

7A. Has there been an impact on your participation in your community since learning that your child has a developmental delay / disability? If so, how?

Prompts: Have you been able to attend church events or other activities taking place in your neighbourhood or community?

7B. Why do you think this is?

Section 8. About support needs …

8A. How satisfied are you with the current support you receive?

8B. Are there any support that do not exist, but you feel will benefit you?

Prompts: What support do you think are necessary for other people in your situation?
