# Supplementary material for: “You don’t expect a miracle to happen soon”: a qualitative study of psychosocial support needs of caregivers of children with disability in Eastern Uganda
Source: BMC Pediatr. 2026 Apr 23;26:533. doi: 10.1186/s12887-026-06906-3 (PMC13245006; doi:10.1186/s12887-026-06906-3)
Supplement: Supplementary file 2 — Additional file 2. [file 12887_2026_6906_MOESM2_ESM.pdf]

# Interview guide for healthcare providers in semi-urban eastern Uganda

## *Respondents*

Staff who regularly provide care at the outpatient paediatric neurology clinic of Jinja Regional Referral Hospital in Jinja City, eastern Uganda and are present on a Wednesday during the 2-week interview period in February and March 2024

## *Objective*

To explore healthcare providers' perceptions of how primary caregivers take care of children with neurodevelopmental disability and to understand their perceptions of carers' psychosocial support needs

## *Introduction*

We are exploring carers' support needs. Through these interviews, we would like to explore healthcare providers' perspectives on the psychological support required by carers of children with neurodevelopmental disability.

## *Questions*

### *Section 1. Healthcare providers' perspectives on caregivers' struggles*

1A. In your experience, what are the challenges primary caregivers may face in taking care of their children?

Prompts: What kind of struggles have you noticed? Do you notice any of them experiencing financial difficulties, overwhelming workload, physical illness or emotional distress?

1B. Do you see any behaviours in caregivers that may affect their physical or emotional health? If so, could you please tell me about them.

Prompts: Are their family members and community supportive? Are there any beliefs or cultural values that may lead to them blaming themselves for their children's neurodevelopmental disability?

### *Section 2. Psychosocial support needs for primary caregivers*

2A. What type of help and support do you think is needed by primary caregivers?

Prompts: What kind of support services do you think the primary caregivers currently require to overcome the struggles they are facing?

2B. What services are currently available to help and support primary caregivers?

2C. Do you think that primary caregivers are aware of and understand how to utilise these services?

2D. How satisfied do you think the caregivers are with the current help and support available?

Prompts: Do you feel that the caregivers are receiving the support they need? Do you think they are able to access the services without any restrictions or hesitation?

2E. Are there areas where current help and support can improve?

Prompts: What type of support is currently lacking? Which area, such as financial difficulties, overwhelming workload, physical illness or emotional distress, needs more support? Do you have any opinions on new support that should be added to the current service?

## *Section 3. Healthcare providers' support for primary caregivers*

3A. What resources and support networks are there for you, as a healthcare provider, to help these children and their primary caregivers?

Prompts: Do the current services provide any guidance or information for healthcare providers to provide support for caregivers' physical and mental health as well as struggles within the household of children with NDD?

3B. Are there areas where current resources and support networks can improve?

Prompts: What type of resources and support networks for healthcare providers is currently lacking? Do you have any opinions on new support that should be added to the current service?
